# A Second Life for MAP, a Model Amphipathic Peptide

**DOI:** 10.3390/ijms23158322

**Published:** 2022-07-28

**Authors:** Sara Silva, Kaido Kurrikoff, Ülo Langel, António J. Almeida, Nuno Vale

**Affiliations:** 1 OncoPharma Research Group, Center for Health Technology and Services Research (CINTESIS), Rua Doutor Plácido da Costa, 4200-450 Porto, Portugal; saracpsilva21@gmail.com; 2 Faculty of Pharmacy, University of Porto, Rua de Jorge Viterbo Ferreira 228, 4050-313 Porto, Portugal; 3 Research Institute for Medicines (iMed.ULisboa), Faculty of Pharmacy, University of Lisbon, Av. Prof. Gama Pinto, 1649-003 Lisboa, Portugal; aalmeida@ff.ulisboa.pt; 4 Institute of Technology, University of Tartu, Nooruse 1, 50411 Tartu, Estonia; kaido.kurrikoff@ut.ee (K.K.); ulo.langel@dbb.su.se (Ü.L.); 5 Department of Biochemistry and Biophysics, Stockholm University, 10691 Stockholm, Sweden; 6 Department of Community Medicine, Information and Health Decision Sciences (MEDCIDS), Faculty of Medicine, University of Porto, Rua Doutor Plácido da Costa, s/n, 4200-450 Porto, Portugal; 7 CINTESIS@RISE, Faculty of Medicine, University of Porto, Alameda Professor Hernâni Monteiro, 4200-319 Porto, Portugal

**Keywords:** model amphipathic peptide, cell-penetrating peptides, delivery system, drug repurposing, CNS, oncology, nanoparticles

## Abstract

Cell-penetrating peptides (CPP) have been shown to be efficient in the transport of cargoes into the cells, namely siRNA and DNA, proteins and peptides, and in some cases, small therapeutics. These peptides have emerged as a solution to increase drug concentrations in different tissues and various cell types, therefore having a relevant therapeutic relevance which led to clinical trials. One of them, MAP, is a model amphipathic peptide with an α-helical conformation and both hydrophilic and hydrophobic residues in opposite sides of the helix. It is composed of a mixture of alanines, leucines, and lysines (KLALKLALKALKAALKLA). The CPP MAP has the ability to translocate oligonucleotides, peptides and small proteins. However, taking advantage of its unique properties, in recent years innovative concepts were developed, such as in silico studies of modelling with receptors, coupling and repurposing drugs in the central nervous system and oncology, or involving the construction of dual-drug delivery systems using nanoparticles. In addition to designs of MAP-linked vehicles and strategies to achieve highly effective yet less toxic chemotherapy, this review will be focused on unique molecular structure and how it determines its cellular activity, and also intends to address the most recent and frankly motivating issues for the future.

## 1. Introduction

Cell-penetrating peptides (CPP) are characterized by an elevated content of basic amino acid residue with a maximum length of 30 amino acid residues. CPPs are unique peptides due to their capability to penetrate biological barriers or membranes without compromising the membrane integrity. Over the years, extended studies have demonstrated that CPP can translocate different biological barriers and successfully deliver a wide range of molecules without cell toxicity and no direct immunological response. The studies showed that CPP can be used as a safe strategy to overcome drug penetration limitations and allows treatment of resistant cancer cell lines [1]. Frankel et al. were the first, in 1988, to discover HIV-Tat protein, and then Vives et al. discovered the key domain for the rapid penetration of this peptide [2,3]. CPPs can be divided into different categories depending on the origin (protein-derived, synthetic, and chimeric CPP), conformation (linear or cyclic) and physicochemical properties (cationic, amphipathic and hydrophobic CPP) [4].

The positive charge of cationic CPPs demonstrated strong electrostatic interactions with negative cytoplasmic membranes that lead to cellular internalization. These CPP sequences often present increased numbers of positively charged arginines and contain more than five positively charged amino acids [5]. There are few hydrophobic CPPs, but still their sequences possess an increased number of non-polar residues (such as alanine, leucine, proline, valine and more) and a few charged amino acids. The most common CPPs discovered are amphipathic CPPs, which contain both non-polar and polar amino acid regions, and often adopt a secondary structure and bind to the membranes depending on the overall conditions [4,6].

The mechanism of cellular uptake is still a delicate subject and has raised a lot of controversy over the years. The different CPP structure features (such as hydrophobicity, peptide charge, hydrophobic moment, and the size of hydrophilic and hydrophobic domain) [7], functionalized cargo and physiological conditions, such as cell type and sample handling (washing, fixation, delays) can influence greatly the mode of penetration that a specific peptide could have [8,9].

The first experimental problem observed was the over-quantification of peptide uptake, and the intra-cellular localization of the peptide. Soon observed that the experimental fixation procedure had an issue and resulted in a wrong hypothesis about direct translocation of the peptides [10]. Another problem encountered was related to the cell sort in flow cytometry with overestimation of the uptake, as at the time the authors were not able to distinguish between membrane-bound peptides and peptides inside the cell [10,11]. Therefore, with these problems in mind before 2003, the main route of CPP penetration was through direct translocation of cell membranes. The authors started to question the previous results only after they found the inherent fixation protocol error when compared to the results obtained in live cellular assays, which arose from the existence of other endocytic mechanisms involved in CPP penetration [11]. Therefore, after 2003, the main mechanism of CPP penetration was re-evaluated and new results were observed. Over the years, research demonstrated that multiple mechanisms of CPP penetration could be involved depending on different environmental, cellular, and structural conditions [12]. In addition, the research conducted also demonstrated that CPP uptake could promote several effects on cell surface, such as curvature changes, modifications in membrane domain architecture, fusion of vesicles, non-bilayer disorder and lipid flip-flop alterations [13]. Today, the mechanisms of cellular uptake can be divided into energy-independent direct penetration and energy-dependent endocytosis [14].

In order to evaluate direct cellular translocation, specific experimental conditions can be used, such as submitting cells to low temperatures, energy depletion with ATP reduction and applying endocytic inhibitors. The first step of CPP penetration starts with initial electrostatic interaction between negatively charged membrane components and positively charged CPP, which can lead to CPP uptake. Different model mechanisms have been proposed to explain cellular CPP penetration, such as transient pore formation or membrane destabilization [15,16]. Transient pore formation, or barrel-stave pores, was first described by Gazit et al. in 1994 [17]. This mechanism is characterized by an initial interaction of CPPs with the cell surface that allows CPPs to assume an amphipathic α-helix structure with basic residue interaction. After this surface interaction, CPPs penetrated the cell bilayer, with the hydrophobic region interacting with the lipid core and the hydrophilic region facing the interior of the transient pore, and forming a barrel-like structure. On the other hand, membrane destabilization is divided in two possible models: the inverted micelle model and the carpet model. The first was studied and proposed by Derossi et al. when describing the pAntp CPP mode of penetration [18]. The inverted micelle model proposes that CPP interaction with the membrane promotes a migration of negatively charged phospholipids to the site, which allows the formation of an inverted micelle, and subsequently the release of CPPs into the intracellular compartment. The carpet model has a different mechanism concept, which describes positively charged amphipathic CPPs distributed horizontally through the membrane surface by binding the acid phospholipids head groups to the cell membrane. This crescent ‘’carpet-like’’ fashion of deposition leads to the gradual destabilization of the cell membrane and successful uptake of CPPs [15].

Energy-dependent endocytosis also represents the main of cellular uptake for many CPPs. Endocytosis is characterized by two distinct mechanisms, phagocytosis and pinocytosis. Phagocytosis mainly occurs with large molecules and usually occurs in specific types of cells that can eliminate pathogens, such as macrophages, neutrophils and monocytes. Pinocytosis usually occurs in the cell membrane and can be divided into four different pathways: macropinocytosis, clathrin endocytosis, caveolae/lipid raft-mediated endocytosis, and clathrin- and caveolae-independent endocytosis [19]. These different pathways differ in the endosome size obtained, the nature of the cargo and the mechanism of endosome formation. As stated before, in order to study the mechanisms associated with CPP entrance into the cell and the escape from the endosome into the cytoplasm, different experimental techniques can be tested. First, we can apply specific endocytic inhibitors such as dynasore, chlorpromazine and sucrose (clathrin-dependent endocytosis), amiloride (inhibits macropinocytosis), nystactin (caveolae-mediated), depletion of cholesterol (inhibits lipid-raft endocytosis) and more [6,20,21,22]. The second part is to ensure that our CPPs or CPP-cargo are released from the endosome, reach their target site and execute their main function. When CPPs or CPP-cargo are entrapped into the endosome, rapid release is required in order to not expose the compounds to degradation from lysosomes. The first hypothesis describes that the CPPs can escape endosomes through pH decrease, which leads to CPP interaction with endosome membranes and subsequently release. Another model states that positively charged CPPs simply tighten the membrane to a point of disruption with interaction of negatively charged components in the endosome [23]. Still, different compounds have been developed to help endosomal escape, such as the lysosomotropic agent chloroquine; the introduction of histidine moieties in the CPP sequence results in a “proton sponge effect” and enhances the osmotic pressure inside the endosome [24]. Moreover, insertion of KLA, GALA, and KALA sequences in the CPP sequence have demonstrated that this promotes endosomal escape [25].

**Figure 1:**
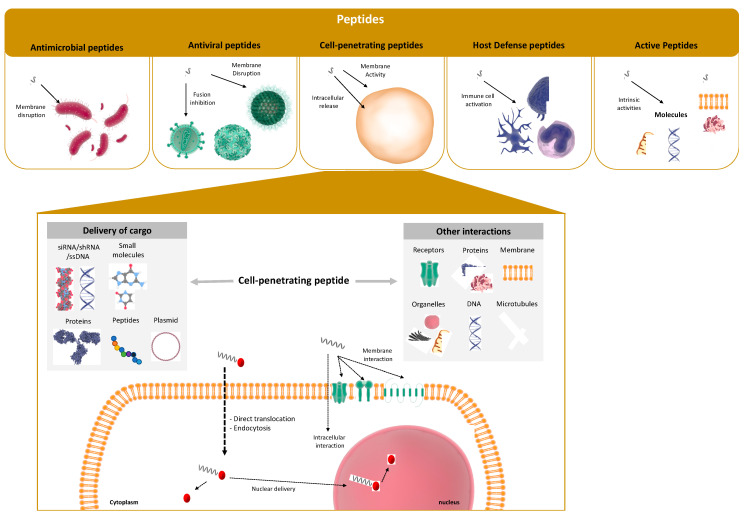
Schematic representation of the different types of peptides available and their different activities. In-depth representation of cell-penetrating peptides as delivery molecules and potential other interactions.

The current research demonstrated a new side of CPPs that can assume other cellular functions, rather than simply being a penetration delivery carrier, but which also presents some intrinsic activity and interaction with important proteins, receptors and modified metabolic activity in cells (Figure 1) [26,27,28,29,30].

**Figure 2:**
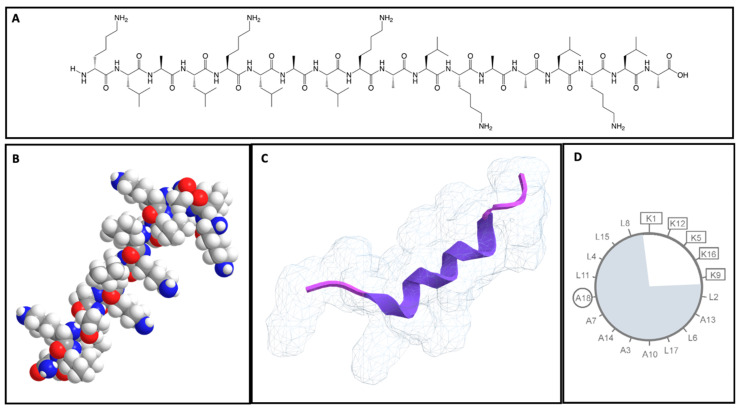
Different representations of peptide MAPs. (**A**)—Structure sequence of MAP (ChemDraw 19.0); (**B**)—Three-dimensional representation of MAP sequence (ChemBio 3D ultra); (**C**)—Secondary structure (in carton) and an H_2_O mesh surface of MAP (3D data retrieved from CPP database (CPPsite 2.0) and analyzed by ChemBio 3D ultra); (**D**)—Projection wheel of MAP (hydrophobic part is blue-shaded and charged residues are highlighted with rectangles).

One CPP that has demonstrated this possibility for dual functionality is the model amphipathic peptide (MAP—KLALKLALKALKAALKLA). The MAP is considered a secondary amphipathic CPP [31] and was first designed by Steiner et al. to study interactions with lipidic interfaces (Figure 2) [32]. Then, in 2005, a pharmaceutical company, Nastech Pharmaceutical Company Inc., unaware of its existence, discovered a peptide, PN159, with the same sequence as a MAP, and demonstrated incredible cellular membrane interactions [33,34,35]. The structure of a MAP allows positioning of hydrophilic and hydrophobic residues on opposite sides, which, in the right conditions, can adopt a stable α-helix structure, getting close to lipidic membrane in temperatures between 25 and 55 °C [36,37], and even a β-structure with increased pressure and concentration [7,38]. In addition, MAPs are characterized by an overall positive net charge of five [39] and are very stable at physiological conditions associated with slow blood clearance (half-life above 72 h) in in vivo studies [40]. The authors demonstrated that arginine and arginine bonds (RR bonds) could lead to degradation susceptibility of peptides, thus showing that peptides not containing RR bonds demonstrated more stability that those with RR bonds in the sequence [40]. All of these features allow MAPs to possess strong cellular penetration in different cell types. Several studies have been conducted to understand and identify the crucial parts of its unique structure that enable its mechanism of cellular penetration and action. Initial studies indicated that amphipathic structure was crucial for its high interaction and penetration of cellular membranes [36,41,42]. Further ahead, new studies with different MAP derivatives demonstrated that both amphipathic and non-amphipathic peptides were able to transgress cell membranes within a few minutes [12,43,44,45]. A study demonstrated that a reduction of four amino acid residues in MAPs, on both the N (KLAL) and C terminal end (LKLA), resulted in a substantial reduction of cell uptake, showing that these four amino acids are crucial for an α-helix to form. Moreover, it was also demonstrated that substitution of five lysines with glutamine residues lead to a reduced uptake and membrane toxicity [36,44], indicating that an α-helix is a structural requirement of MAPs to contribute to higher membrane interaction and higher cellular uptake. The MAP fluorescent pattern observed indicated that both non-endocytic and endocytic modes of cell uptake are involved due to cytosol and nucleus accumulation, accompanied by a punctuate pattern around the nucleus [43,44,46,47]. Another interaction observed was that MAPs had the ability to interact with cellular tight junctions and modulate tight junctions (TJ) to enhance systemic delivery [35]. TJs are composed of three main proteins (occluding, claudins and junctional adhesion molecules) and are important for promoting the integrity of the cell barrier, and their dynamic structure regulates endocytic processes [35]. In recent years, this capability of MAPs was further studied by Bocsik et al., and several experiments were conducted in order to understand which TJ-specific target was involved. The authors demonstrated that 10 µM for 1 h did not cause significant change in the metabolic activity of Caco-2 cell lines or brain endothelial cells, and showed an increased molecular permeability through both barriers after MAP incubation [48]. The authors, based on affinity-binding measurements, discovered that MAPs were most effective as a modulator of junctional permeability acting via claudin-1 and -5 [48]. In addition, in a more recent study it was demonstrated that MAPs successfully interacted with TJs, promoting enhanced permeability of different compounds (such as quinidine and verapamil). The authors also showed a concentration-dependent effect of MAPs with 3 µM, resulting in significant changes in the claudin-1 pattern and the actin cytoskeleton organization. These results allow the understanding that MAPs can act on claudins-1, -4 and -7 and open cell–cell junctions. Through dock analysis, the authors observed that Q44 and V55 residues of claudin-1 and -7 can form a binding pocket for L11 of the peptide, and lysine K1 and K5, and leucine L6 and L17 interacted with all three claudins tested [37].

Besides the cellular delivery capabilities of MAPs, other features and properties have been associated with this peptide. Kilk et al. observed that MAPs could potentially promote long-term effects on the metabolic profile of cells [29]. Another particularity of MAPs is their strong lytic capabilities at concentrations above 4 µM, and high aggregation propensity when close to lipidic membranes [49,50,51].

In this review, we will focus on the current studies conducted with MAPs, their own unique capabilities, and their utility in different research areas.

## 2. MAPs as Delivery Carrier

The importance of new alternative delivery mechanisms is increasing as a consequence of current drug limitations, such as first-passage metabolism, secondary toxic effects, off-target therapeutic effects, tumor resistance, solubility and biological barrier penetration. This general systemic distribution leads to the need for higher drug therapeutic concentrations to reach the target spot. In the ideal scenario, it will be possible to administer the minimal drug dosage required to have therapeutic activity only at the target site without compromising non-target organs, reducing side-effects and healthcare costs [52]. However, this may also enable the delivery of different molecules that require a carrier to reach cells, such as antisense oligonucleotides, peptides, proteins, dyes, siRNAs, etc. [53]. The use of CPPs as delivery molecules allows several advantages over conventional techniques.

Several articles demonstrated high cellular uptake of fluorescent dye covalently linked with MAPs inside different cell lines [41,46,54,55,56]. These studies were conducted to evaluate the mechanisms of cellular uptake used by MAPs when interacting with cells. For instance, the results indicate that multiple processes of cellular uptake might be involved with MAP penetration [41], and at least 50% uptake was found even with incubation temperatures below 0 °C on calf aortic endothelial cells [46]. The use of confocal laser scanning microscopy allowed detection of MAPs evenly distributed throughout the cytoplasm and nucleus accompanied by a punctuate pattern near the nucleus [43,44]. It also demonstrated that MAP penetration abilities were consistent in the different cell lines tested [44]. Moreover, the work of Säälik et al. showed that MAPs accumulate into giant plasma membrane vesicles (GPMVs) lumen, both at room- as well as at low temperature. The use of GPMVs and giant unilamellar vesicles (GUVs) have been shown to be an ideal model to analyze the translocation of CPPs across the plasma membrane in conditions lacking endocytosis and the role of lipids for translocation [57,58,59]. The authors demonstrated that amphipathic CPPs efficiently uptake into the membranes that are partially depleted of cholesterol or that are less ordered. The addition of cholesterol resulted in an inhibitory effect on the cellular uptake of MAPs. In the case of MAPs, the lower fluorescence intensity from cholesterol-rich vesicles suggests that the interaction with lipids, and especially with less densely packed membrane zones, is of a different nature compared to non-amphipathic CPPs [55]. The authors further explored this result in other papers, and showed that enhanced cholesterol content and tighter packing of membranes predominantly decrease the accumulation of MAPs in vesicles, indicating that the uptake of MAPs takes place preferentially via the more dynamic membrane regions [56].

Another type of delivery compound was used by Oehlke et al. covalently conjugated with MAPs. The authors decided to attach a peptide nucleic acid (PNA) to MAPs and evaluate delivery efficiency, since this antisense agent is characterized by its poor cellular uptake. The results show that MAP conjugation lead not only to 20-fold enrichment of PNA in cells, but also contributed to enhanced cellular response directed against the mRNA of the nociceptin/orphanin FQ receptor [54]. Furthermore, cytochrome C (Cyt-c) was conjugated with MAP in two ways (reducible disulfide or a non-reducible thioether linkage) and determined its nuclear delivery ability. Cyt-c is an essential molecule responsible for initiating programmed cell death and apoptosis when Cyt-c in the cytosol is rapidly degraded by proteasome, and its inhibition could lead to accumulation of Cyt-c and elicit an apoptotic response. The authors demonstrated that the conjugation of Cyt-c with CPP-R8 or MAPs increased the amount of protein by 4-fold and 26-fold, compared to unmodified Cyt-c in the HEK293 cell line, respectively. Similar results were obtained for HeLa cells with a 6-fold and 38-fold increase, respectively. In addition, the authors demonstrated that MAPs conjugated successfully delivered Cyt-c to the nucleus [60] and elicited 19.6% apoptosis in cells when compared with 11.7% of non-treated cells in proteasome-inhibited cells [47]. The conjugate also remains stable even with reach of the nucleus compartment. The authors also confirm that the vesicle fusion mechanism is crucial to MAP conjugate nuclear internalization, so there is a close implication of endocytosis in nuclear transport [47,60].

## 3. MAP’s Intrinsic Activity

MAPs appear to possess more than CPP function depending on the concentration applied in study. The dual activity of MAPs appears to promote cellular long-term effects and even promote cell death with an increase of concentration. For instance, cytosolic metabolic alterations on CHO cells were observed with MAP treatment [29]. Furthermore, it appears that conjugation of MAPs with other active compounds enhances its activity against different diseases in in vitro models, such as neurodegenerative diseases and cancer. For example, in a study conducted by Vale et al., it was shown that conjugation of rasagiline, a propargylamine of monoamide oxidase B (MAO-B), with MAPs resulted in a decrease in the percentage of cells with α-syn inclusions at a lower concentration. The results suggested that both MAPs and rasagiline could possibly interact with α-syn, inhibiting its capability to form inclusions and inducing a neuroprotective effect [61].

Several α-helical peptides have demonstrated their activity as membrane permeate agents in host defense systems, and direct disruption or pore formation are the main mechanisms of action present in these peptides. The moment that the membrane is compromised, electrochemical potential is lost and cellular cytoplasmic homeostasis disappears, and cell death occurs. Still, the membrane perturbation energy depends not only on the peptide used but also on the composition and nature of the lipid mixture present in the cell membranes. Different peptide behaviors will reflect on different pore sizes and insertion thresholds observed [62]. It is important to mention that bacterial and tumor cell membranes share common features, such as: a negatively charged membrane and high transmembrane potentials [51]. Tumor cells contain three to up to seven times more phosphatidylserine than normal cells, and is located mainly in the outer leaflet, which results in a more negatively charged outer membrane. On the other hand, normal cells have more neutral membrane and low membrane potentials composed of phosphatidylcholine, sphingomyelin, phosphatidylethanolamine, cholesterol and zwitterionic phospholipids. Phosphatidylserine is localized exclusively in the inner leaflet of the membranes [63,64]. All these different features can allow peptides to selectively interact more with tumor cell lines rather than normal cells [65]. The antiproliferative activity against different tumor cell lines (breast cancer MCF-7 and neuroblastoma SH-SY5Y) was observed with MAPs conjugated with tacrine. The authors observed a decrease of 50% cell viability at a concentration of 2.5 µM for SH-SY5Y and 5 µM for MCF-7, and with loss of morphological appearance and high cell death at 20 µM [66]. The same authors further observed an increase in the antiproliferative effect of MAPs conjugated with the central nervous system (tranylcypromine and rasagiline) against neuroblastoma cell line SH-SY5Y and colorectal adenocarcinoma Caco-2 cell lines when compared to MAPs alone and a MAP derivate (Lys(N_3_)-MAP) [67]. In a different work, the toxic activity was also observed by Moutal et al., with treatment of MAPs conjugated with Ca^2+^ channel binding domain 3 (CBD3) on cortical neuron cultures. The authors demonstrated that treatment with MAP-CBD3 lead to an increased influx of Ca^2+^ and nuclear targeting, consequently inhibiting the desired neuroprotection activity [30]. Anti-bacterial activity was also assessed by Bocski et al., demonstrating that MAPs presented an antimicrobial effect at low concentrations against different ESKAPE pathogens [37].

## 4. Functionalization of MAPs into Nanoparticles

Targeted intracellular delivery is a challenging strategy with the need for several molecules to be involved to work properly. Taking this into consideration, the use of CPPs has demonstrated great potential in functionalizing nanoparticles which might allow the remedy of several limitations present in each mechanism individually. Successful data have shown that conjugation can be beneficial to a new potential therapeutic approach against different diseases [68]. In a study carried out by Zaro et al., a pH-sensitive CPP nanoconstruct was constructed and compromised by a highly pH-sensitive co-oligopeptide sequence fused to MAPs to prevent non-target internalization and disguise the cationic charge. The pH sensitivity was acquired by the incorporation of histidine residues that allowed the peptide to be internalized at pH 6 instead of pH 7.4 [69]. The decrease of pH in extracellular media surrounding tumors was observed, and this strategy could allow a more targeted approach to tumors [70,71]. The authors fused MAPs with glutathione S-transferase protein and a 10-mer histidine moieties oligopeptide sequence. The results demonstrated a pH-dependent surface binding and internalization to cell membrane [68]. This nanoparticle and MAP association was further evaluated in two complementary works conducted by Silva et al. First, it was demonstrated that the encapsulation of conjugated Tacrine-MAP into lipid nanoparticles lead to a decrease in cell viability against two cancer cell lines, SH-SY5Y and MCF-7, compared to treatment with free Tacrine-MAP conjugate [66]. In the following work, the authors evaluated two more conjugates, tranylcypromine-MAP and rasagiline-MAP, encapsulated into the same lipid nanoparticles in vitro. The results demonstrated that incorporation of both conjugates into nanoparticles led to a significant decrease in cell viability at lower concentrations on both cell line studies (SH-SY5Y and Caco-2) when compared to the conjugates administered alone [67]. These results demonstrated the potential use of this strategy in new therapeutic approaches, reducing the amount of drug use and increasing the therapeutic efficacy. Furthermore, other nanoparticle functionalization alternatives were evaluated by Silva et al. with lysine-modified MAPs and Tacrine-MAP conjugate adsorption into lipid nanoparticle surfaces. The authors determined that both peptides appeared to interact with the surface of nanoparticles, creating electrostatic interactions in a concentration-dependent matter. The analysis indicated that adsorption of lysin-modified MAPs and Tacrine-MAPs promoted a significant increase in zeta potential values independent of whether NLC was loaded with a drug or not. In addition, the authors evaluated the adsorption capabilities of more physiological states (PBS pH 7.4), incubating both peptides and nanoparticles. The data showed an increase in particle size, polydispersity index and zeta potential, suggesting a more pronounced adsorption with the formation of a multilayer on the nanoparticle surface, or a possible peptide–peptide interaction resulting in nanoparticle aggregation [72].

## 5. In Silico Studies Involving MAPs for New Directions in Biomedicine

**Figure 3:**
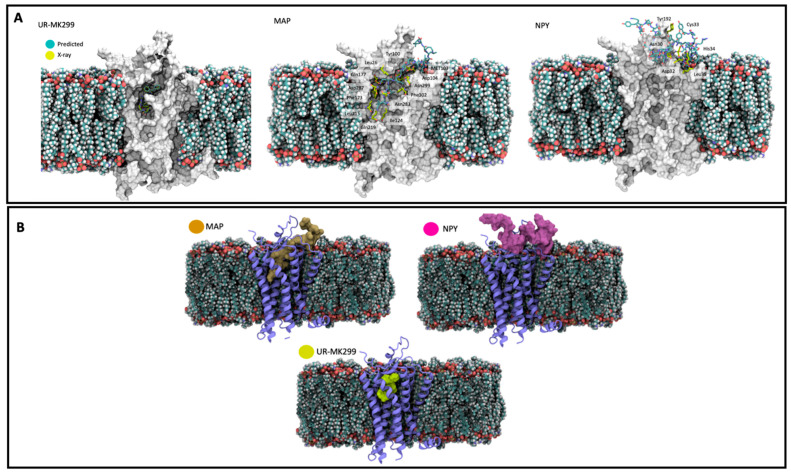
(**A**)—Slab-view interaction with UR-MK299, MAP and NPY against NPY1R obtained by molecular docking with information obtained by X-ray diffraction. (**B**)—Representation of the volume occupied by UR-MK299, MAP and NPY when complexed with NPYR1 (Reproduced and edited from [71], MDPI, 2022).

The use of in silico methods is exponentially growing when applied to pharmacology. These new approaches allow the prediction and suggestion of hypotheses, and provide discoveries through information uploaded in in silico models or in simulation programs [73]. Several studies have demonstrated a success with the use of computer-assisted drug design in the discovery of new mechanisms or structure-based drugs [74,75]. For instance, molecular docking is becoming more accurate and widely used, due to rapid improvements in computational platforms and the number of protein–ligand complexes available. Docking in silico experiences determine a possible mechanism of molecular recognition and validate a possible mode of ligand–receptor interaction. Different programs can be applied for molecular docking simulations of large data bases, such as AutoDock Vina, DOCK, FlexX, GOLD, and ICM [76]. This type of approach led to the discovery that MAP potential has a strong association with Neuropeptide y receptor 1 (NPYR1) when compared to the control UR-ML299 antagonist used, and even Neuropeptide Y. Neuropeptide Y is a 36-amino-acid neuropeptide, is highly expressed on central and peripheral neurons, and can act as a neurotransmitter or neuromodulator which displays multiple functions, including modulating neurogenesis and neurotrophins, decreasing excitotoxicity, regulating calcium homeostasis, and attenuating neuroinflammation [77,78,79]. Neuropeptide Y interacts with neurons through the binding and activation of different receptors such as Y1, Y2, Y4, Y5, and Y6 that are coupled with G-proteins [80]. The data demonstrated that MAP has a deep insertion into the NPYR1 cavity through N-terminal amino acid residues (Figure 3) [72]. It is important to highlight that both MAPs and neuropeptide Y can form an α-helix structure that could be essential for the receptor interaction observed. More in silico studies are needed to understand a possible MAP interaction with these receptors and the main physiological cellular outcomes that can arise from this interaction. These receptors could be beneficial in neurodegenerative diseases once NPY is seen as important to regulating neuroprotection. A possible interaction with MAPs could lead to a neuronal response depending on if it is inhibiting or activating the receptor.

## 6. Conclusions

The research performed so far has demonstrated that MAPs can assume different properties depending on the type of application. This review showed that amphipathic peptide MAPs can easily penetrate cell membranes by multiple mechanisms, including pore formation and endocytic pathways. In addition, the data show that MAPs can reach the nucleus, are highly stable and can form aggregates. The concentration-dependent behavior allows, at higher concentrations, lytic activity and easy adsorption into nanoparticle surfaces. Covalent conjugation of MAPs with other compounds was introduced and the results show the potential use of this conjugation in cancer in vitro models. Furthermore, promising in silico molecular docking data show a new potential feature of MAPs with receptor interaction. Still, some hemolytic activity has to be observed and more studies need to be exploited to understand if modifications in MAP sequences could be beneficial [81,82,83] and to open new ways of addressing new cellular interactions. Until today, no clinical trial was implemented with the use of MAPs. Even with these promising activities, MAPs still require more safety analysis in more complex models.

## Data Availability

Not applicable.
